# Supplementary material for: LANA-Dependent Transcription-Replication Conflicts and R-Loops at the Terminal Repeats (TR) Correlate with KSHV Episome Maintenance
Source: bioRxiv. 2025 Mar 13:2025.03.10.642343. Preprint. [Version 1] doi: 10.1101/2025.03.10.642343 (PMC11952399; doi:10.1101/2025.03.10.642343)
Supplement: Supplement 1 [file NIHPP2025.03.10.642343v1-supplement-1.pdf]

bioRxiv preprint doi: https://doi.org/10.1101/2025.03.10.642343; this version posted March 13, 2025. The copyright holder for this preprint (which was not certified by peer review) is the author/funder, who has granted bioRxiv a license to display the preprint in perpetuity. It is made available under aCC-BY 4.0 International license.

**Supplementary Materials**

**Supplementary Fig. S1.** Colocalization of epigenetic marks with LANA at KSHV TR. **A.** Schematic of the KSHV genome showing the terminal repeats (TR) relative to the unique region open reading frames (blue) and primer positions for TR, ORF45, and ORF75. **B.** ChIP-qPCR for histone H3K4me3, H3K27ac, H3K9me3, LANA or control IgG assayed at the TR, ORF45 or ORF75 loci in BCBL1 or iSLK cells. **C.** Same as in panel B, except ChIP antibodies with RAD21, CTCF, or IgG control. ** $p<.01$, *** $p<.001$, student 2-tailed t-test, n=3 biological replicates.

**Supplementary Fig. S2.** R-loop formation at the KSHV TR. A. DRIP assay with BC1 cells using S9.6 (blue) or control IgG (black) assayed with primers for cellular actin or KSHV TR ORF45 and ORF75. *** $p<.001$, student two-tailed t-test. B. IGV screen shot of RNA transcripts mapped to KSHV TR region using public data sets for total RNAseq in BCBL1 cells during latent conditions (SRR15069589, SRR15069590, SRR15069591). The reference map consists of a

bioRxiv preprint doi: https://doi.org/10.1101/2025.03.10.642343; this version posted March 13, 2025. The copyright holder for this preprint (which was not certified by peer review) is the author/funder, who has granted bioRxiv a license to display the preprint in perpetuity. It is made available under aCC-BY 4.0 International license.

small region of the unique region with K15 and 2 copies of the TR. TR transcripts Tr(1) and Tr(2) are indicated above.

**Supplementary Fig. 3. Colocalizations of LANA, H3pS10 and PCNA.** Representative images of iSLK cells showing the percentage of cells with LANA and colocalizations with H3pS10 and PCNA. 60x magnification, N=4, total of 126 cells, ****p<.0001, student two-tailed t-test.

**Supplementary Fig. S4. Confocal microscopy IF analysis of H3pS10 and LANA colocalization in iSLK cells.** H3pS10 (blue), LANA (red), Dapi (blue).

**Supplementary Fig. S5. Confocal microscopy IF analysis of PCNA-H3pS10-LANA colocalization in iSLK cells.** PCNA (green), H3pS10 (blue), LANA (red).

**Supplementary Fig. 6. Western blot of BCBL1 cells treated with FVP or triptolide. A-B.** Western blots of BCBL1 cells treated with 0.4 mM FVP (pane A) or with 2 mM triptolide (panel B) for 0, 2, 4 or 24 hrs and probed for LANA, RNAPII pS2, pS5, H3pS10, or b-actin.

**Supplementary Movie. M1. Confocal microscopy IF analysis of PCNA-H3pS10-LANA colocalization in iSLK cells.** PCNA (green), H3pS10 (blue), LANA (red).

# Supplementary Figure 1

bioRxiv preprint doi: https://doi.org/10.1101/2025.03.10.642343; this version posted March 13, 2025. The copyright holder for this preprint (which was not certified by peer review) is the author/funder, who has granted bioRxiv a license to display the preprint in perpetuity. It is made available under aCC-BY 4.0 International license.

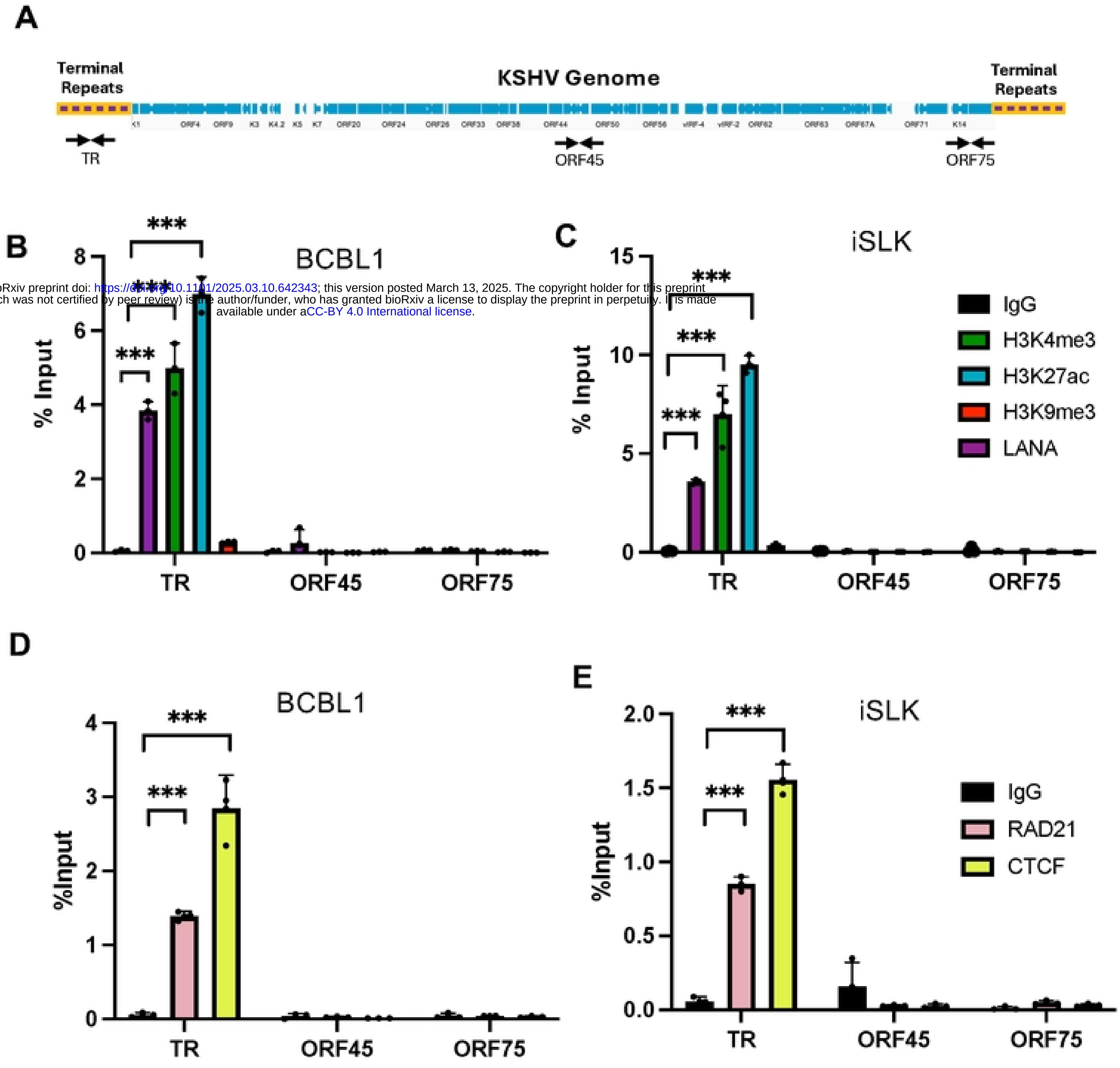

# Supplementary Figure 2

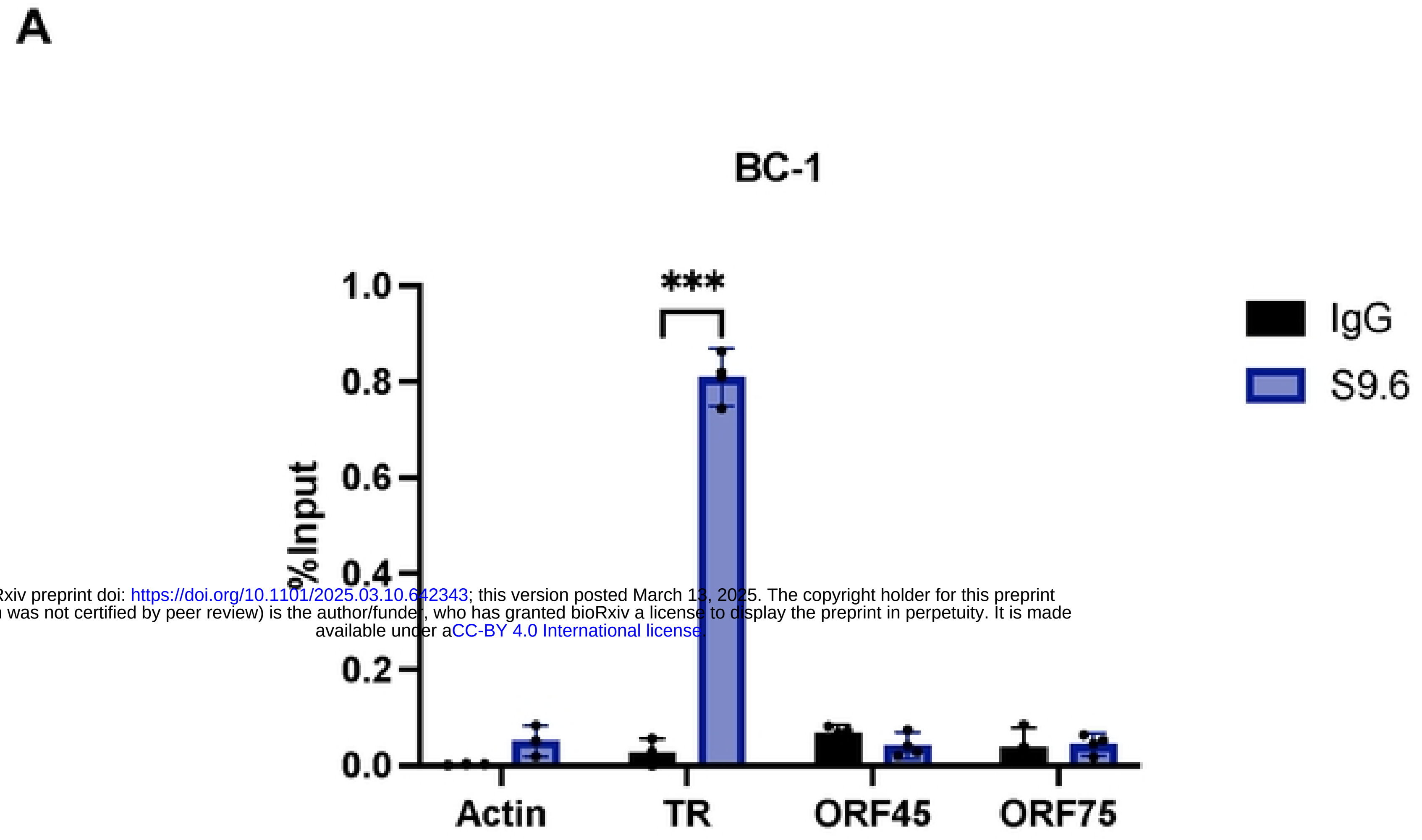


bioRxiv preprint doi: https://doi.org/10.1101/2025.03.10.642343; this version posted March 13, 2025. The copyright holder for this preprint (which was not certified by peer review) is the author/funder, who has granted bioRxiv a license to display the preprint in perpetuity. It is made available under aCC-BY 4.0 International license.

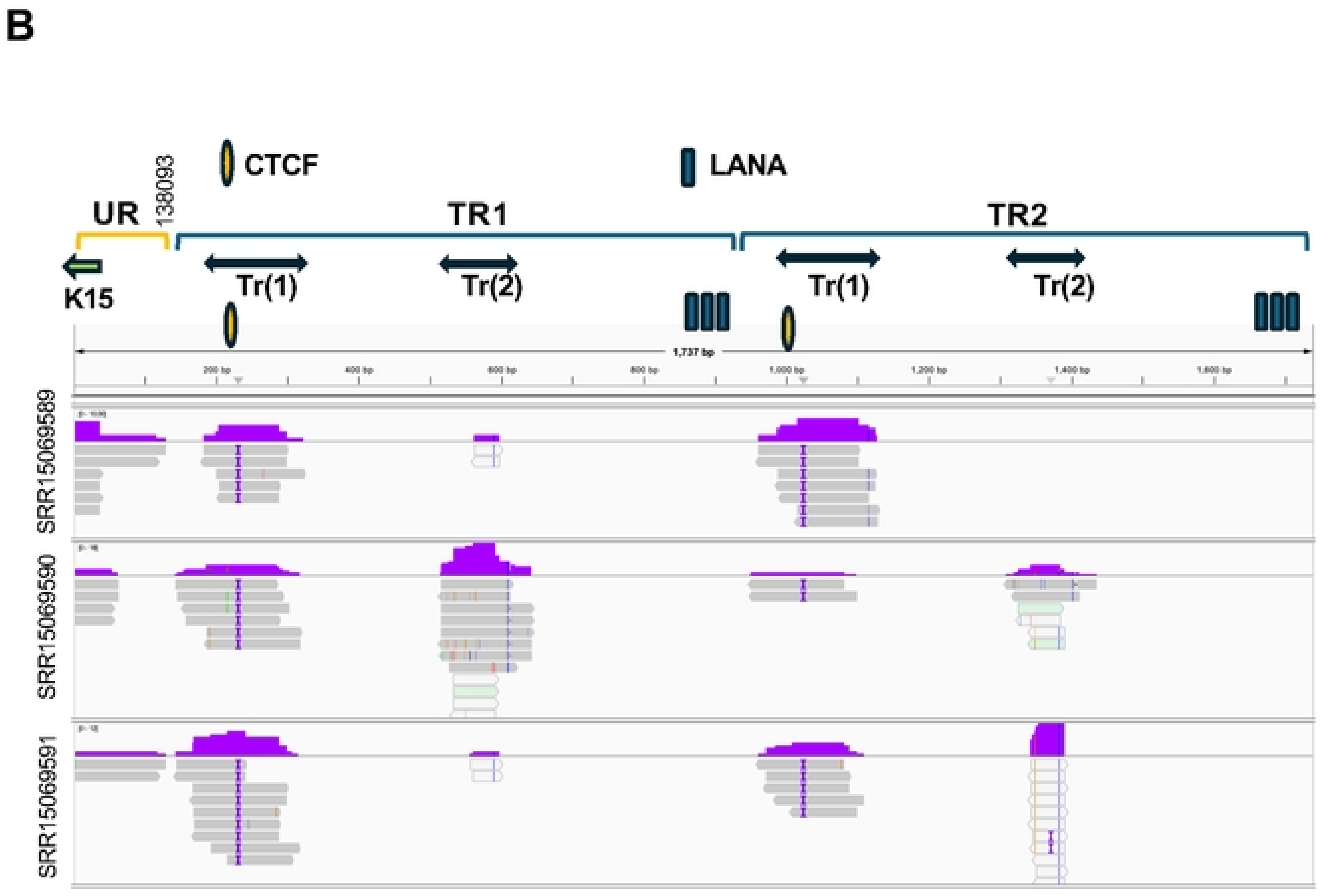

Supplementary Figure 3

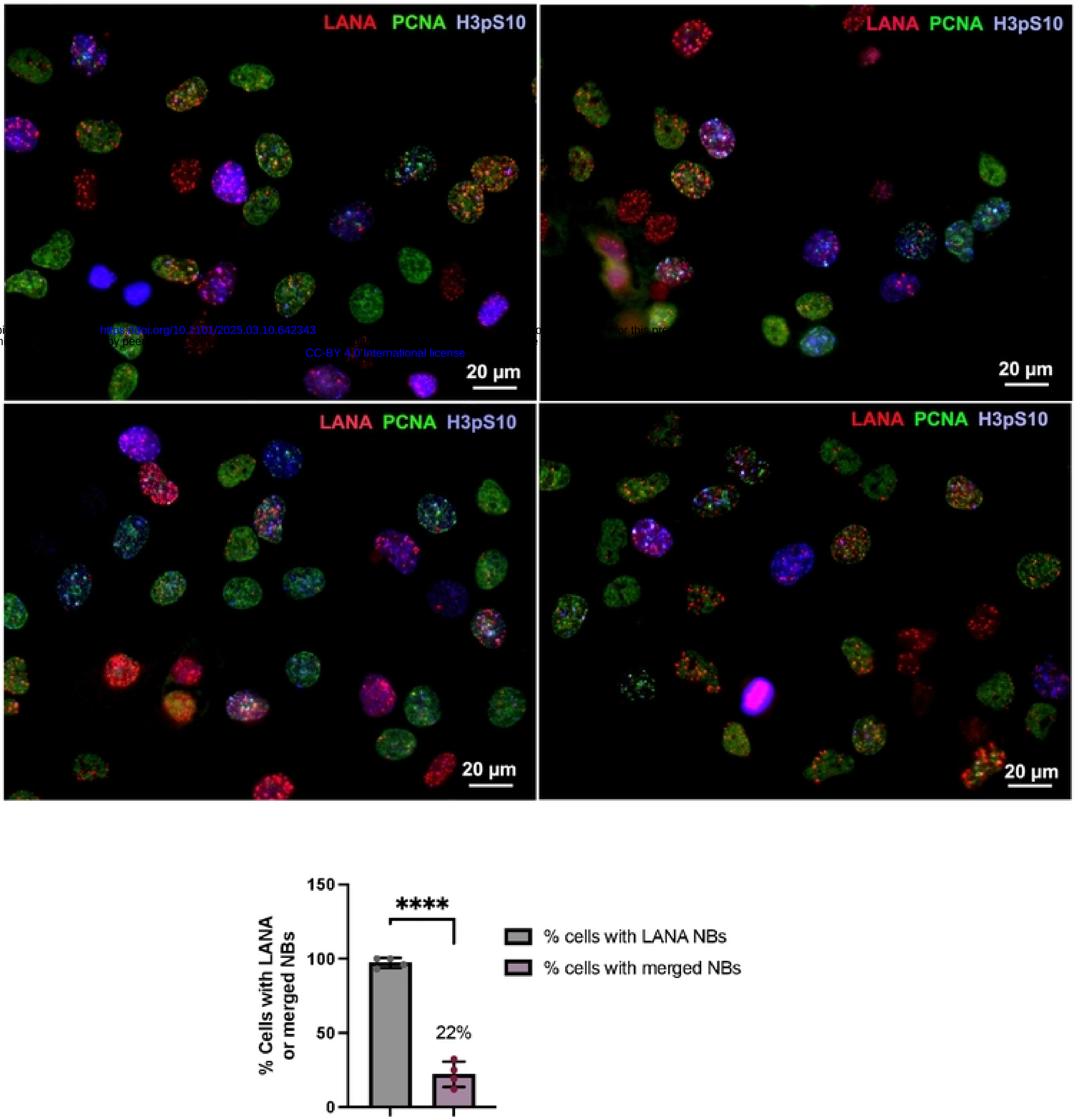


https://doi.org/10.1101/2025.03.10.642343
CC-BY 4.0 International license

# Supplementary Figure 4

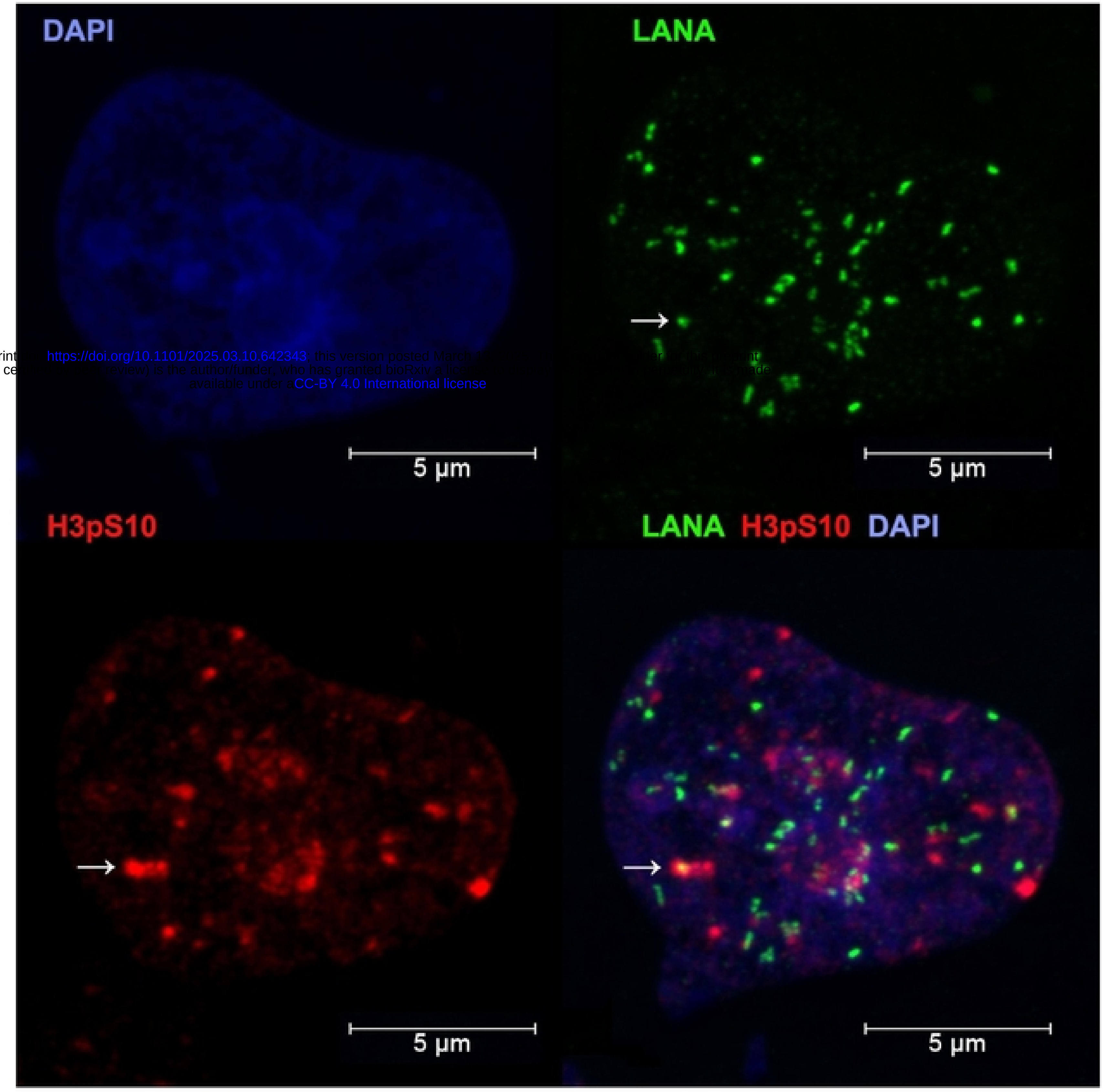


bioRxiv preprint [illegible] https://doi.org/10.1101/2025.03.10.642343; this version posted [illegible]
(which was not ce[illegible] who has granted bioRxiv a [illegible]
[illegible] CC-BY 4.0 International license

# Supplementary Figure 5

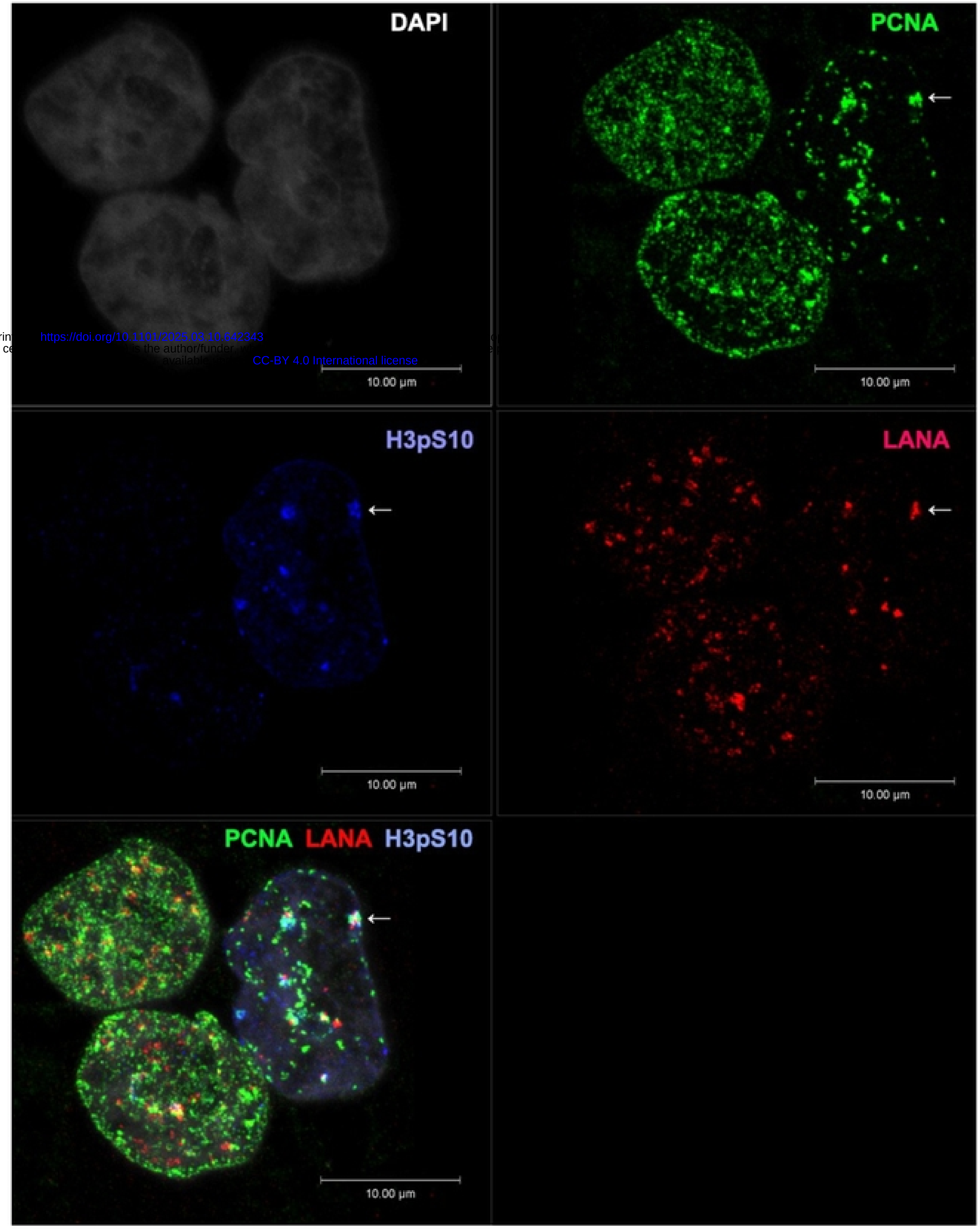


bioRxiv preprin[illegible] https://doi.org/10.1101/2025.03.10.642343 [illegible]
(which was not ce[illegible] the author/funder [illegible]
available [illegible] CC-BY 4.0 International license

# Supplemental Figure 6

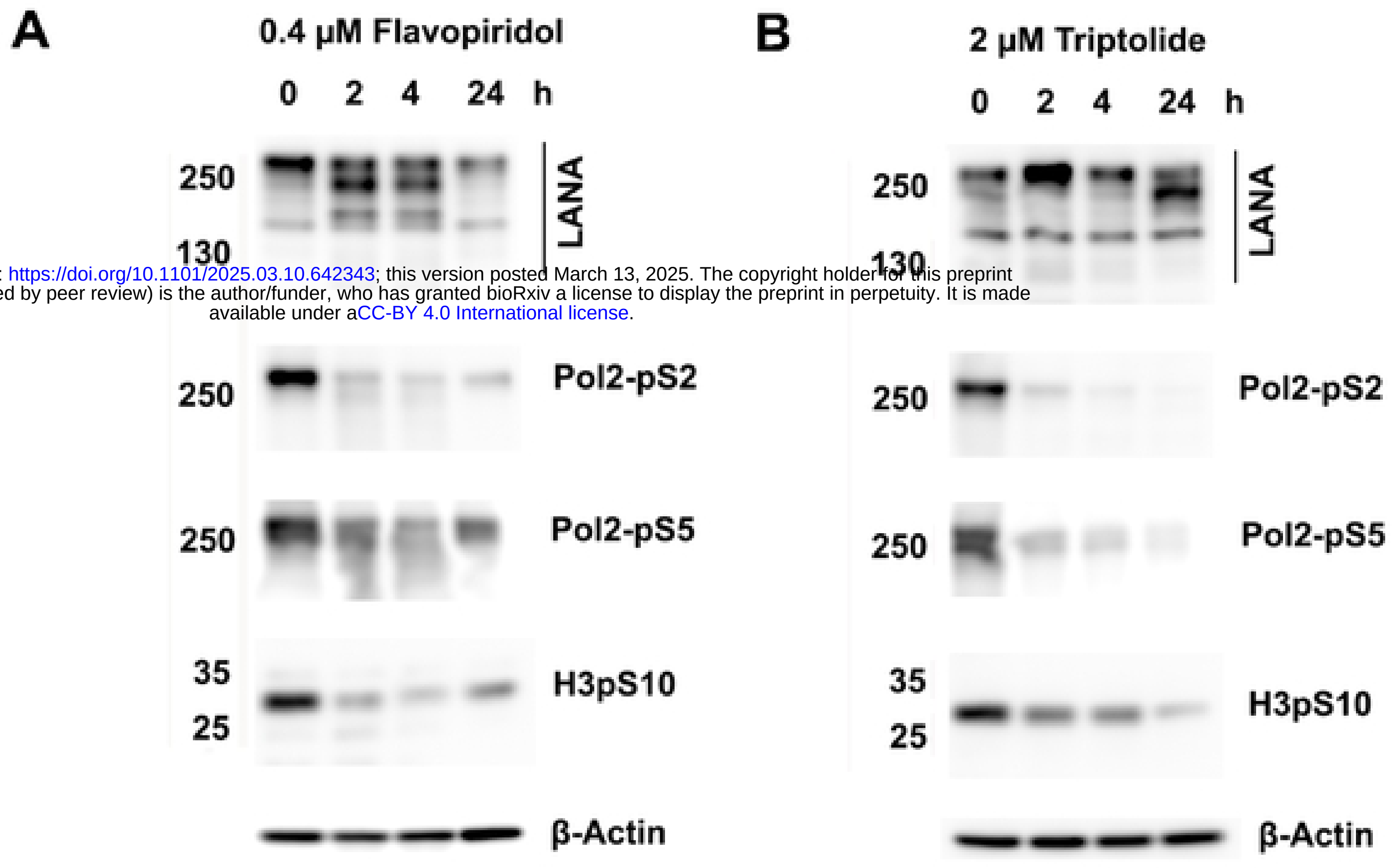


bioRxiv preprint doi: https://doi.org/10.1101/2025.03.10.642343; this version posted March 13, 2025. The copyright holder for this preprint (which was not certified by peer review) is the author/funder, who has granted bioRxiv a license to display the preprint in perpetuity. It is made available under aCC-BY 4.0 International license.

bioRxiv preprint doi: https://doi.org/10.1101/2025.03.10.642343; this version posted March 13, 2025. The copyright holder for this preprint (which was not certified by peer review) is the author/funder, who has granted bioRxiv a license to display the preprint in perpetuity. It is made available under aCC-BY 4.0 International license.

Supplemental Movie 1. Confocal image of H3pS10, PCNA, LANA
